# Epidermal wound healing in severe sepsis and septic shock in humans

**DOI:** 10.1186/cc7932

**Published:** 2009-06-24

**Authors:** Marjo Koskela, Fiia Gäddnäs, Tero I Ala-Kokko, Jouko J Laurila, Juha Saarnio, Aarne Oikarinen, Vesa Koivukangas

**Affiliations:** 1 Department of Anesthesiology, Division of Intensive Care Medicine, Oulu University Hospital, Kajaanintie 50, BOX 21, 90029 OUH, Finland; 2 Department of Surgery, Oulu University Hospital, Kajaanintie 50, Oulu, BOX 21, 90029 OUH, Finland; 3 Department of Dermatology, Oulu University Hospital, Kajaanintie 50, Oulu, BOX 21, 90029 OUH, Finland

## Abstract

**Introduction:**

The effect of sepsis on epidermal wound healing has not been previously studied. It was hypothesised that epidermal wound healing is disturbed in severe sepsis.

**Methods:**

Blister wounds were induced in 35 patients with severe sepsis and in 15 healthy controls. The healing of the wounds was followed up by measuring transepidermal water loss and blood flow in the wound, reflecting the restoration of the epidermal barrier function and inflammation, respectively. The first set of suction blisters (early wound) was made within 48 hours of the first sepsis-induced organ failure and the second set (late wound) four days after the first wound. In addition, measurements were made on the intact skin.

**Results:**

The average age of the whole study population was 62 years (standard deviation [SD] 12). The mean Acute Physiology and Chronic Health Evaluation II (APACHE II) score on admission was 25 (SD 8). The two most common causes of infections were peritonitis and pneumonia. Sixty-six percent of the patients developed multiple organ failure. The decrease in water evaporation from the wound during the first four days was lower in septic patients than in the control subjects (56 g/m^2 ^per hour versus 124 g/m^2 ^per hour, *P *= 0.004). On the fourth day, septic patients had significantly higher blood flow in the wound compared with the control subjects (septic patients 110 units versus control subjects 47 units, *P *= 0.001). No difference in transepidermal water loss from the intact skin was found between septic patients and controls. Septic patients had higher blood flow in the intact skin on the fourth and on the eighth day of study compared with the controls.

**Conclusions:**

The restoration of the epidermal barrier function is delayed and wound blood flow is increased in patients with severe sepsis.

## Introduction

Sepsis and systemic inflammatory response syndrome have been assumed to disturb epidermal barrier function and wound healing [1-3]. Sepsis has profound effects on the maintenance of epithelial barriers: the barriers of gut and gall bladder, which are essential to homeostasis and innate immune function, have been shown to be disturbed by sepsis and multiple organ dysfunction syndrome [3-5]. Sepsis has been clinically associated with wound infections and disturbed anastomotic and fascial healing [3]. Furthermore, in animal models, leukocyte infiltration into a wound site has been shown to be diminished in sepsis [2]. The process of wound healing requires a well-orchestrated network of inflammation, cell proliferation, migration, and protein synthesis, which may be disturbed by inflammatory surge [6]. Septic patients often need surgical interventions, and impaired healing can lead to various complications. Understanding the mechanisms of impaired wound healing could therefore enable improvements in treatment.

With the suction blister model, two essential parts of wound healing can be studied: the restoration of the epidermal barrier function and blood flow reflecting the level of inflammation in the wound [7]. The suction blister model has been previously used for studying the basic biology of epidermal wound healing and the healing of burn injuries as well as the effect of jaundice and diabetes on epidermal wound healing [8-11]. In this model, a prolonged vacuum induces the disruption of the dermo-epidermal junction and separates the epidermis from the dermis while the basal lamina remains intact. Epidermal proliferation takes place from the edge and migration covers the defective area [1,7]. The restoration of barrier integrity can be followed by measuring transepidermal water loss (TEWL) (the decrease in water loss) [12]. Blood flow in the wound can be studied using laser Doppler flowmetry [13].

The aim of this study was to assess the restoration of epidermal barrier function and blood flow in blister wounds in severe sepsis. Skin water evaporation and blood flow in severe sepsis were compared with those in healthy controls. Our hypothesis was that water evaporation in healing blister wounds is increased in severe sepsis because of the delayed epidermal barrier restoration. We also assumed that wound blood flow may be disturbed in severe sepsis.

## Materials and methods

### Patients

The study was approved by the local ethics committee. Written informed consent was obtained from each patient or next of kin. The setting was a 12-bed medical-surgical intensive care unit (ICU) at the Oulu University Hospital. Patients were treated according to the normal ICU protocol and current severe sepsis guidelines [14], including hydrocortisone supplementation in septic shock refractory to vasopressor therapy.

All patients admitted from 9 May 2005 to 15 December 2006 with sepsis were considered eligible for the study. Standard definitions were used to define sepsis, severe sepsis, and septic shock [15]. Exclusion criteria included age under 18, any bleeding disorder, immunosuppression therapy, surgery not related to the sepsis, surgery during the preceding 6 months, malignancy, chronic hepatic failure, chronic kidney failure, and steroid treatment not related to sepsis.

The following information was collected from all study patients: age, gender, reason for admission to the ICU, focus of infection, severity of underlying diseases on admission as assessed by the Acute Physiology and Chronic Health Evaluation II (APACHE II) and Simplified Acute Physiology Score II (SAPS II) scores, evolution of daily organ dysfunctions assessed by daily sepsis-related organ failure assessment (SOFA) scores, presence of ischaemic heart disease, chronic obstructive pulmonary disease (COPD), diabetes mellitus, and asthma. The lengths of stay in the ICU and in hospital were recorded as well as the ICU, hospital, and 30-day mortalities. For controls, we used 15 healthy Caucasian age-matched volunteers (7 men and 8 women).

### Experimental blister wound

We used the suction blister device to create experimental wounds of standard size as described earlier [7,16]. The study outline is presented in Table 1. The first set of experimental wounds was induced within 48 hours from the first sepsis-induced organ failure (early wound). A suction blister device (Mucel Ky, Nummela, Finland) with five 8-mm-diameter bores was applied to the intact abdominal skin and connected to the vacuum pump, which created a negative pressure on the area. First we used a higher vacuum (about 60 to 70 kPa) and after 20 to 30 minutes a lower one (40 to 50 kPa). During blister induction, the warming of the skin accelerates blister formation. The blister roofs were removed after induction. We used the same device for both patients and controls. A second set of wounds (late wound) was induced 4 days after the first set of wounds (Table 1). One set of suction blisters was induced in the controls.

**Table 1:** The course of the study

Study days	0^a^	1	2	3	4	5	6	7	8
	First suction blister induction				Second suction blister induction				
					
	Early wound								
	
					Late wound				
					
Measurements	Blood flow and water evaporation				Blood flow and water evaporation (from both wounds)				Blood flow and water evaporation

^a^The first samples were obtained within 48 hours from the first organ failure.

### Measurements

The restoration of epidermal barrier function was followed up by measuring water loss from the blister wound. Since the epidermal barrier is a tightly regulated gateway to a percutaneous passage, TEWL decreases when the epidermal barrier is restored [17]. After blister induction (when there is no epidermis), the water evaporation is 15- to 20-fold higher than in the intact skin. During the healing process, evaporation decreases, enabling the non-invasive follow-up of epidermal healing [7,8,18,19]. In this study, TEWL was measured using a VapoMeter (Delfin Technologies Ltd, Kuopio, Finland) [20], which measures the amount of water loss in grams per square metre. There is a cylindrical chamber in the head of the VapoMeter where the sensors for humidity and temperature are located. The VapoMeter forms a closed chamber on the skin in which the system automatically calculates the evaporation rate from the increase in relative humidity. The VapoMeter has been shown to be a reliable device to measure barrier function [21,22]. All five blister wounds were measured and the mean value was calculated and reported. We also measured TEWL from the intact abdominal skin while simultaneously measuring that of the blister wounds.

A laser Doppler flow meter (Periflux Pf1; Perimed KB, Stockholm, Sweden) was used to measure the blood flow in the blister wounds and also in the intact abdominal skin [23,24]. The laser beam penetrates about 1 mm into the skin. The vasculature of the skin contains two plexuses but the laser Doppler reaches only the superficial one, which lies just beneath the dermo-epidermal junction [17,25]. All five blister wounds were measured and the mean was calculated and reported. The measurements are expressed as perfusion units, which is arbitrary. Measurements of TEWL and skin blood flow in the blister wounds were taken twice on each set of wounds: the first measurements following the induction of the wound and the second measurements on the fourth day of healing (Table 1). All blister wounds were covered with an air- and water vapour-permeable, self-adhesive dressing between the study days (Mepore; Mölnlycke Health Care AB, Göteborg, Sweden). All blister inductions and measurements were performed by MK and FG under the same circumstances, such as the same air temperature.

### Statistical analysis

The data were entered into an SPSS database (SPSS Data Entry, version 3.0; SPSS Inc., Chicago, IL, USA). Summary statistics are expressed as median with 25th and 75th percentiles or as mean with standard deviation (SD), and the analysis between the groups was done using the Kruskal-Wallis test. The Mann-Whitney *U *test was applied to analyse the differences between the two groups. The categorical variables were analysed by Fischer exact test. Spearman correlation was calculated. Two-tailed *P *values are reported and the analyses were performed by the SPSS software (version 15.0; SPSS Inc.). The differences were considered significant at *P *values of less than 0.05.

## Results

### Patient characteristics

Two hundred sixty-three patients with sepsis were screened during the study period. Consent was obtained from 44 patients who fulfilled the inclusion criteria of severe sepsis. The first set of blisters could be induced in 35 patients within 48 hours of detection of the first organ failure and these 35 patients were included in the final analysis. The later blister wound was induced in the patients who were in hospital and alive on day four (27 patients, 77%). The last measurements were taken when the patients were still hospitalised, on the eighth day of the study. Table 2 summarises the clinical characteristics and the co-morbidities of the patients. There was no difference in average age between the septic patients and controls.

**Table 2:** Baseline characteristics of the study patients

	Septic patients
Male gender	22 (63%)
Age, years	62 (range 24 to 80, SD 12)
Body mass index, kg/m^2^	28 (range 21 to 47, SD 6)
Co-morbidities	
Diabetes mellitus	9 (26%)
COPD	4 (11%)
Asthma	3 (9%)
Ischaemic heart disease	7 (20%)
Focus of infection	
Lungs	15 (43%)
Intra-abdominal	12 (34%)
Urinary	1 (3%)
Primary blood	2 (6%)
Other	5 (14%)
Severity of the disease	
APACHE II score on admission	25 (range 9 to 44, SD 8)
SOFA score	9.3 (range 1 to 22, SD 4)
Sepsis steroid	24 (69%)
Septic shock	29 (83%)
Multiple organ failure	23 (66%)
Outcome variables	
ICU length of stay, days	7.9 (range 2 to 30, SD 6)
ICU mortality	7 (20%)
Hospital mortality	9 (26%)
30-day mortality	10 (29%)

APACHE II, acute physiology and chronic health care II; COPD, chronic obstructive pulmonary disease; ICU, intensive care unit; SD, standard deviation; SOFA, sepsis-related organ failure assessment.

### Restoration of the epidermal barrier (transepidermal water loss)

**Figure 1:**
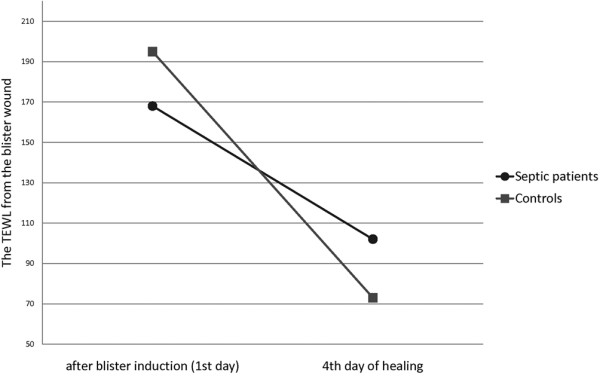
The decrease of transepidermal water loss (TEWL) from the first to the fourth day of the early wound. The decrease was lower in the septic group compared with controls (*P *= 0.004).

During epidermal healing, the TEWL from the blister wound decreases. The decrease reflects the restoration of the epidermal barrier function. The decrease of TEWL from day 0 to day 4 in the early wound was lower in the septic group than in the control one (Figure 1, Table 3). The mean decreases were 56 g/m^2 ^per hour (SD 91) in the septic group and 124 g/m^2 ^per hour (SD 31) in the control group. The same trend was seen in the late wound, for which the decreases were 77 g/m^2 ^per hour (SD 63) in the septic group and 124 g/m^2 ^per hour in the control group (*P *= 0.091) (Table 3). This suggests that the restoration of the epidermal barrier function is diminished in severe sepsis.

**Table 3:** Water loss from the wound

Group	Day 0 of the early wound		Day 4 of the early wound		Day 0 of the late wound		Day 4 of the late wound	
				
	TEWL	SD	TEWL	SD	TEWL	SD	TEWL	SD
Septic	168	58	102	93	180	88	86	45
Controls	195	30	73	31	195	30	73	31
	*P *= 0.087		*P *= 0.484		*P *= 0.424		*P *= 0.590	

Values other than *P *values are expressed in grams per square metre per hour. SD, standard deviation; TEWL, transepidermal water loss.

There were no differences in the TEWL of the intact skin at any point in time between the septic patients and the control subjects. On day 0, the mean TEWL values from the intact abdominal skin were 14 g/m^2 ^per hour (SD 17) in the septic group and 10 g/m^2 ^per hour (SD 7) in the control group. Neither the wound nor the intact skin showed any difference in TEWL between patients who received or did not receive steroid treatment due to sepsis.

### Blood flow in the blister wound

**Figure 2:**
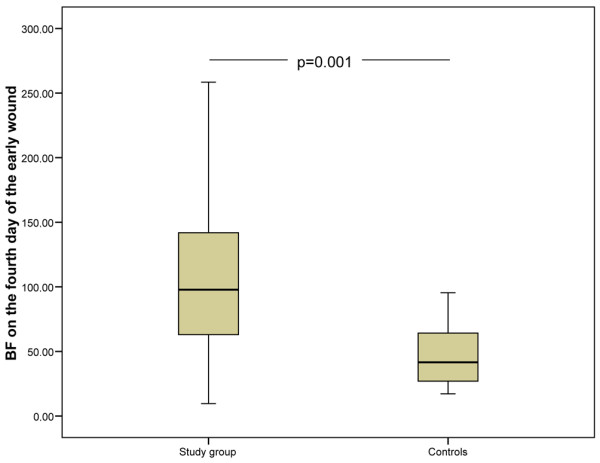
Skin blood flow on the fourth day of the early wound. A significant difference was found between the control group and the study group. The lower and upper edges of each box indicate the interval between the 25th and 75th percentiles. The vertical line represents the range and the horizontal line within the box represents the median of each group. BF, blood flow.

**Figure 3:**
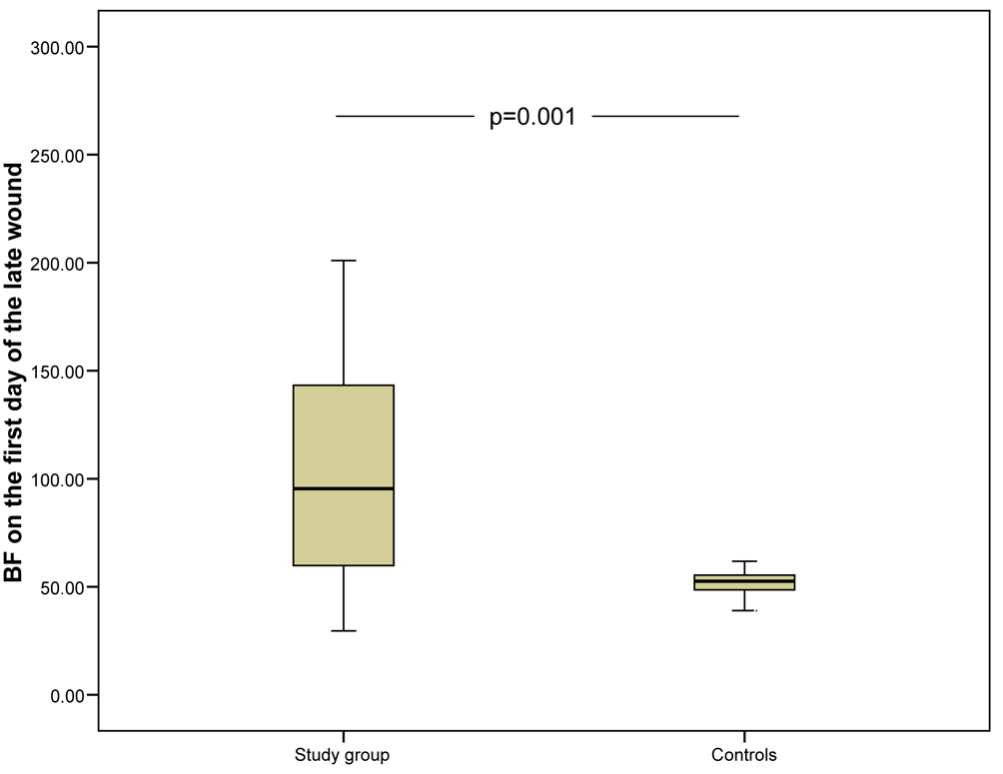
Skin blood flow on the first day of the late wound. A significant difference was found between the study group and the control group. The lower and upper edges of each box indicate the interval between the 25th and 75th percentiles. The vertical line represents the range and the horizontal line within the box represents the median of each group. BF, blood flow.

**Figure 4:**
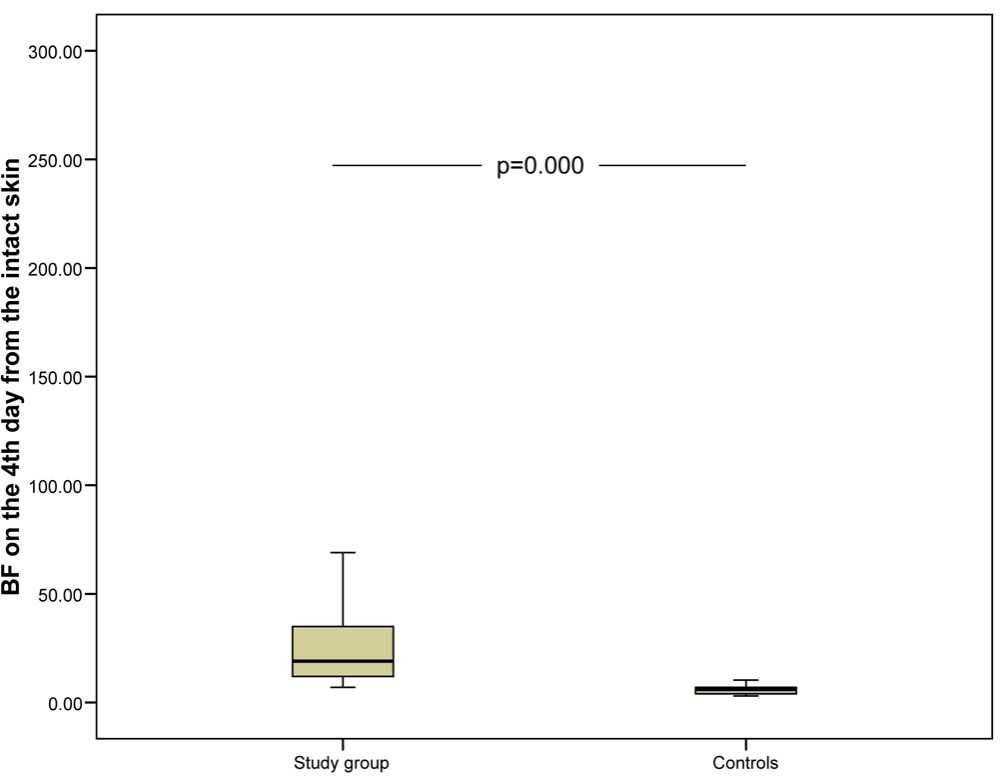
Skin blood flow on the fourth day of the study from the intact skin. The study group had significantly higher blood flow than the control subjects. The lower and upper edges of each box indicate the interval between the 25th and 75th percentiles. The vertical line represents the range and the horizontal line within the box represents the median of each group. BF, blood flow.

In the early wound, there were no differences in blood flow after blister induction between groups (Table 4). This suggests that the initial inflammation does not show any alteration in the early phase of sepsis. On the contrary, the mean blood flow on the fourth day of healing in the early wound was significantly higher in the septic group, which suggests that sepsis aggravates the healing-related induction of inflammation (Figure 2 and Table 4). In the late wound, the mean blood flow after blister induction was significantly higher in septic patients than in the control subjects (Figure 3). It was also higher on the fourth day of healing (eighth day of the study) (Table 4). This suggests that both initial and induced wound inflammation are intensified in patients with established septic disease, which is possibly the result of systemic inflammation. The blood flow values did not differ between patients who received or did not receive steroid treatment. On the first day, there were no differences in mean blood flow from the intact abdominal skin in the septic group (15 units, SD 12) and in the control group (14 units, SD 9). However, on the fourth day, the mean blood flow from intact skin was higher in the septic group (24 units, SD 18) compared with the controls (6 units, *P *= 0.000) (Figure 4).

**Table 4:** Blood flow from the blister wound

Group	Day 0 of the early wound		Day 4 of the early wound		Day 0 of the late wound		Day 4 of the late wound	
				
	BF	SD	BF	SD	BF	SD	BF	SD
Septic	76	49	110	67	101	50	110	58
Controls	51	11	47	24	51	11	47	24
	*P *= 0.273		*P *= 0.001		*P *= 0.001		*P *= 0.005	

Values other than *P *values are expressed in perfusion units. BF, blood flow; SD, standard deviation.

## Discussion

We found that the restoration of the epidermal barrier was delayed in patients with severe sepsis compared with the control subjects. This was seen in both the early and the late stages of disease. We also found that wound blood flow was more pronounced in patients with sepsis when compared with the control group. As hypothesised, the septic patients had delayed epidermal wound healing. Blood flow response was enhanced in sepsis, which possibly arose from high systemic inflammation [26,27].

Previous studies on wound healing in sepsis have focused on wound collagen synthesis. Overall, previous data suggest that sepsis disturbs wound connective tissue synthesis [28-30]. There are no previous data concerning epidermal cell kinetics and inflammation in wound healing in septic patients.

We made experimental wounds to our patients by using a suction blister device. This wound model is non-invasive and safe to the patient. Therefore, it was applicable even in the case of critically ill patients. The suction blister device causes a standard-sized epidermal wound allowing accurate comparisons between individuals. This model provides information about the recovery of skin barrier functions and wound blood flow. The parameters observed are physiological: water loss from the wound reflects restoration of the epidermal barrier, and blood flow in the wound mirrors inflammation.

Epidermal barrier function and its restoration were evaluated by measuring TEWL [12,31,32]. The decrease in water loss from the wound reflects the restoration of the epidermal barrier function [22]. When critically ill patients in the ICU are studied, the prevalent conditions such as patient temperature, ambient temperature, fluid balance, and administration of vasopressor drugs must be taken into consideration. In this study, there was no correlation between temperature, fluid balance, or noradrenalin dose and water evaporation. Water evaporation was measured by using a closed chamber system. With a closed chamber system, the effect of external or body-induced air flows can be avoided [33]. It is also possible that some increase in water evaporation from the wound is the result of increased capillary permeability in sepsis. However, the control subjects had higher water evaporation after blister induction than the septic patients, which suggests that increased vascular permeability did not have a notable effect on wound water evaporation.

Increased wound blood flow (change from normal skin blood flow) in the wound is caused by inflammation [34] and is considered a reliable parameter for overall wound inflammation [17,35]. After wounding, a short vasoconstrictive phase is followed by vasodilatation, which peaks after a few days of healing and then calms down toward final healing [35].

We found that wound blood flow response was higher in the patients with sepsis than in the controls [36,37]. Early sepsis is characterised by hyper-inflammation and excess of pro-inflammatory mediators such as tumour necrosis factor-alpha and interleukin-1 and interleukin-6 [38,39]. It is possible that these mediators cause an increase in local inflammation, as well. Nitric oxide (NO) is a mediator of early wound healing and inflammation [35]. The levels of NO are increased in sepsis [39,40]. The local effects of NO could also be related to increased wound blood flow. The observed delay in restoration of the epidermal barrier in sepsis could be related to increased wound inflammation and suppressed macrophage function [41]. It has been shown that defects in regulating cytokine expression and abnormally high transforming growth factor-beta-induced inflammation delay wound healing [42,43]. The wound establishes a balance between too little inflammation, which increases the risk of infection, and excessive inflammation, which contributes to disturbed wound healing [42,43]. It is possible that high inflammation in septic patients disturbs epidermal cell proliferation or migration.

## Conclusions

Wounds in septic patients have delayed restoration of the epidermal barrier function and increased local inflammation compared with those of control subjects.

## Key messages

• Epidermal healing is delayed in patients with severe sepsis.

• Initial wound blood flow (that is, initial inflammatory response to trauma) in early septic disease is at the normal level, but induced inflammation during wound healing is aggravated in septic patients, which can be an effect of systemic inflammation on local inflammation.

## Abbreviations

ICU: intensive care unit; NO: nitric oxide; SD: standard deviation; TEWL: transepidermal water loss.

## Competing interests

The authors declare that they have no competing interests.

## Authors' contributions

MK helped make the blister wounds and measurements, drafted the manuscript, and helped perform the statistical analysis. FG helped make the blister wounds and measurements and helped perform the statistical analysis. VK and TIA-K helped draft the manuscript and helped conceive the study and participated in its design and coordination. JJL and AO participated in the design of the study. JS helped conceive the study and participated in its design and coordination. All authors have read and approved the final manuscript.
